# Targeting pepsin provides superior protection to acid suppression in reflux‐associated airway inflammation and lung dysfunction

**DOI:** 10.1113/EP093251

**Published:** 2026-11-05

**Authors:** Thiago Meneses Araújo Leite Sales, Humberto Barbosa da Costa Filho, Kalinne Kelly Lima Gadelha, Kerolayne de Melo Nogueira, Álvaro Xavier Franco, Carlos Eduardo da Silva Monteiro, Maria Klayre Araújo de Sousa, Monalisa Cássias Souza, Isabele de Sá Silveira Melo, Lucas Antonio Duarte Nicolau, Marco Antonio de Freitas Clementino, Pedro Jorge Caldas Magalhães, Alexandre Havt, Pedro Marcos Gomes Soares, Daniel Sifrim, Marcellus Henrique Loiola Ponte de Souza

**Affiliations:** ^1^ Department of Nursing Estácio University Center of Ceará Fortaleza Ceará Brazil; ^2^ Department of Clinical Medicine, Faculty of Medicine Federal University of Ceará Fortaleza Ceará Brazil; ^3^ Department of Physiology and Pharmacology, Faculty of Medicine Federal University of Ceará Fortaleza Ceará Brazil; ^4^ Laboratory of Inflammation and Translational Gastroenterology (LIGAT) Federal University of Delta do Parnaíba, Parnaíba Piauí Brazil; ^5^ Department of Morphology, Faculty of Medicine Federal University of Ceará Fortaleza Ceará Brazil; ^6^ Barts and the London School of Medicine and Dentistry Queen Mary University of London London UK

**Keywords:** gastro‐oesophageal reflux disease, lung inflammation, pepsin, pepstatin

## Abstract

Gastro‐oesophageal reflux disease is a prevalent condition associated with extra‐oesophageal manifestations, such as pulmonary inflammation. Pepsin, an essential gastric enzyme, has been implicated in pulmonary complications owing to its inflammatory effects. Although acid‐suppressive therapies, such as proton pump inhibitors, reduce gastric acidity, they do not prevent reflux episodes, microaspiration or the harmful effects of pepsin. This study evaluated the effects of pepsin inhibition by pepstatin on inflammation and pulmonary dysfunction in a murine model of reflux. Male Swiss mice were anaesthetized with ketamine and xylazine and subjected to a surgical procedure involving pyloric stenosis and complete stenosis of the gastric fundus to induce reflux. The mice were divided into four groups: sham, reflux, reflux + pepstatin and reflux + omeprazole. Treatments were administered daily for 28 days. The evaluated parameters included oesophageal and pulmonary wet weights, myeloperoxidase activity, cytokine levels [interleukin (IL)‐6, IL‐10 and keratinocyte chemoattractant], histopathological alveolitis scores, functional analyses of spirometry and tracheal contractility. The reflux group exhibited significant oesophageal and pulmonary oedema, increased myeloperoxidase activity, elevated levels of IL‐6 and keratinocyte chemoattractant, reduced IL‐10, severe alveolitis, impaired pulmonary function and tracheal hyper‐reactivity. Pepstatin treatment significantly reduced inflammatory and functional alterations in the reflux model. In contrast, omeprazole was less effective at controlling inflammation and improving pulmonary function. These findings suggest that pepsin inhibition might reduce pulmonary inflammation and help to preserve lung function in a murine reflux model. Targeting pepsin, rather than solely suppressing gastric acidity, shows promise in mitigating reflux‐related extra‐oesophageal complications.

## Abstract (highlights)

- **What is the central question of this study?**
  Pepsin contributes to pulmonary inflammation and respiratory dysfunction in gastro‐oesophageal reflux disease, but the effects on lung outcomes of pepsin inhibition in comparison to acid suppression have not been investigated fully.
- **What is the main finding and its importance?**
  We adapted an experimental murine model of reflux‐induced pulmonary inflammation. Pepsin inhibition with pepstatin significantly reversed pulmonary damage, whereas proton pump inhibitor therapy was ineffective. These findings highlight pepsin as a key mediator of reflux‐associated respiratory dysfunction and support its inhibition as a potential therapeutic strategy.

## 1 INTRODUCTION

Gastro‐oesophageal reflux disease (GORD) is a clinical condition in which gastric contents flow back into the oesophagus, leading to heartburn and regurgitation (Vakil et al., 2006). The main factors contributing to GORD include dysfunction of the lower oesophageal sphincter (LOS), presence of a hiatal hernia, increased frequency of transient LOS relaxation, acidic pocket formation and delayed gastric emptying (Chen & Brady, 2019; Katz et al., 2013).

Scientific evidence indicates that GORD is associated with chronic respiratory diseases, such as idiopathic pulmonary fibrosis, chronic cough and asthma (Durazzo et al., 2020; Robinson & DiMango, 2014). These conditions can arise either from the aspiration of gastric contents or from stimulation of the vagus nerve. Chronic microaspiration of small amounts of gastric contents following reflux has been hypothesized to lead to repeated injury and ultimately contribute to the development of pulmonary fibrosis. Another proposed mechanism involves the activation of acid‐sensitive receptors in the oesophagus, which transmit afferent signals via the vagus nerve. This can trigger reflex constriction not only in the oesophageal smooth muscles but also in the airway structures, thereby contributing to respiratory complications (Broers et al., 2018; Hom & Vaezi, 2013).

Despite these insights, the molecular mechanisms underlying microaspiration of gastric components (i.e., acid, pepsin and bile salts) remain poorly understood. Experimental models have proved valuable tools for investigating these phenomena. Appel et al. (2007) demonstrated that the endotracheal instillation of gastric contents in rats induced an inflammatory response. Previous studies have demonstrated that individual endotracheal administration of acid, bile salts or pepsin to rats leads to differential increases in inflammatory cytokines (Samareh Fekri et al., 2014) and that the microaspiration of gastric components promotes the development of pulmonary inflammation and fibrosis (Chen et al., 2017, 2019). Pepsin, a proteolytic enzyme (Barrett et al., 2012), is a key component of gastric reflux in the airways. Evidence suggests that instillation of pepsin into the lower airways induces significant inflammatory (Samareh Fekri et al., 2014) and fibrotic (Chen et al., 2019) responses in lung tissue. Pepsin has also been identified as a biomarker of gastric pulmonary aspiration and is associated with lung rejection following transplantation (Farrell et al., 2006; Stovold et al., 2007).

Considering the molecular changes induced by the gastric contents, particularly pepsin, debates regarding the effectiveness of acid suppression in preventing tissue injury are ongoing. However, pepsin can retain some enzymatic activity at a pH close to 6.0 and is inactivated at pH levels >6.5 (Johnston et al., 2007). Although proton pump inhibitors (PPIs) modify gastric acidity, they do not decrease the frequency of reflux episodes or microaspiration events, which limits their protective effects against tissue injury (Samuels & Johnston, 2020). Mertens et al. (2010) observed that gastric juice from patients receiving acid‐suppressive therapy provoked an inflammatory reaction in bronchial epithelial cells. This suggests that acid suppression does not fully prevent damage to the pulmonary tissue. Finally, a meta‐analysis revealed that antacid therapies had no robust effect on respiratory outcomes (Khor et al., 2022). These findings highlight the need for further research to develop new therapeutic strategies for pulmonary disorders secondary to GORD.

Owing to the limitations of acid‐suppression therapies, such as PPIs, studies have shown that pepsin inhibition can prevent and reduce cellular injury in oesophageal and extra‐oesophageal tissues exposed to gastric reflux. Nagahama et al. (2006) demonstrated that gastric administration of pepstatin prevented oesophageal ulcers in an experimental oesophagitis model, whereas other studies reported that pepstatin inhibited tonsillar hypertrophy in vitro (Kim et al., 2018) and reduced laryngeal inflammation in laryngopharyngeal reflux models (Johnston et al., 2023). Additionally, aspartic protease inhibitors have been shown to preserve laryngeal and oesophageal mucosal barrier function (Sales et al., 2024, 2026) and to protect against E‐cadherin cleavage and matrix metalloproteinase dysregulation in murine laryngeal and oesophageal cell cultures (Blaine‐Sauer et al., 2023; Samuels et al., 2023). Based on this evidence, the aim of this study was to investigate whether pepsin inhibition by pepstatin or gastric acid suppression by omeprazole could attenuate pulmonary inflammation and rescue impaired lung function in a murine model of gastric reflux.

## 2 MATERIALS AND METHODS

### 2.1 Ethical approval

All experimental procedures were conducted at the Laboratory for the Study of Gastrointestinal Physio‐Pharmacology of the Federal University of Ceará (UFC), in Fortaleza, Ceará, Brazil. The experiments followed the *Guide for the Care and Use of Laboratory Animals* of the US National Institutes of Health (Bethesda, MD, USA). In addition, the study was conducted in accordance with, and adheres to, the animal experimentation policies of *Experimental Physiology*. All procedures were approved by the Ethics Committee on the Use of Animals of the UFC (approval no. 2946300919).

### 2.2 Animals

The sample size was determined a priori based on a power analysis assuming 80% statistical power (α = 0.05) (Berg et al., 2024), while accounting for the expected mortality associated with the experimental model to ensure a final effective sample size of 5–10 surviving mice per experimental group for each analysis. Accordingly, a total of 132 male Swiss mice (30–35 g) were obtained from the Bioterium ‘Professor Eduardo Torres’ of the UFC. Upon arrival, animals were randomly allocated to experimental groups by simple randomization, placed in standard cages in ventilated chambers in controlled conditions (22°C ± 2°C; 12 h–12 h light–dark cycle) and provided with free access to standard chow, water and environmental enrichment.

Animal welfare was assessed daily using a standardized clinical scoring evaluation. Pre‐established humane end points were strictly applied to prevent unnecessary pain or prolonged distress. Criteria for early study discontinuation and immediate humane euthanasia included: (1) progressive body weight loss exceeding 20% compared to pre‐operative baseline; (2) severe lethargy, ataxia, persistent hunched posture or impaired ambulation resulting in the inability to access food or water; (3) marked respiratory distress (severe dyspnoea); or (4) marked lack of responsiveness to mild tactile stimulation or severe hypothermia.

All 26 mice that died prior to the study completion met these pre‐established clinical criteria and were humanely euthanized by anaesthetic overdose [intraperitoneal overdose of ketamine (300 mg/kg, Syntec do Brasil Ltda, Santana de Parnaiba‐Sao Paulo) and xylazine (30 mg/kg, Syntec do Brasil Ltda, Santana de Parnaiba‐Sao Paulo). No animals were found dead spontaneously in their cages in the absence of humane intervention.

### 2.3 Surgical procedure and experimental design

An experimental mouse model of reflux was generated surgically using the method previously described by Silva et al. (2017). After fasting for 12 h, the mice were anaesthetized using an intraperitoneal injection of ketamine (100 mg/kg) and xylazine (10 mg/kg), and a median laparotomy was performed to exteriorize the stomach. The gastric fundus region was completely ligated, and substenosis was subsequently induced in the pyloric region close to the duodenum. In the sham group, only the stomach was exteriorized, without performing the additional surgical procedures. To optimize postoperative recovery and minimize distress during the critical 72 h postsurgical period, animals received subcutaneously administered buprenorphine (0.1 mg/kg) for analgesia, warm sterile 0.9% saline solution (0.5 mL, subcutaneously) for fluid resuscitation, and a highly palatable nutritional dietary gel supplement to support hydration and nutrition. Additionally, environmental enrichment, including red polypropylene shelters and nesting material, was placed in the cages. A total of 106 animals survived the experimental procedures, corresponding to an approximate mortality rate of 20%. Animals that died before the planned euthanasia time points were excluded from subsequent analyses. At the end of the experiments, animals were euthanized by anaesthetic overdose consisting of ketamine (300 mg/kg) and xylazine (30 mg/kg), corresponding to three times the anaesthetic dose used for surgery, administered via intraperitoneal injection. Death was confirmed by the cardiorespiratory arrest, in accordance with the guidelines of the Animal Ethics Committee of the UFC.

Two experimental sets were conducted (Tables 1 and 2). The first set aimed to evaluate the temporal course of the disease. For this purpose, mice subjected to experimental reflux were euthanized on postoperative days 7, 21 or 28. Analyses included wet weight measurement, myeloperoxidase (MPO) assessment of oesophageal and lung tissues, spirometry and tracheal contractility, all performed in a blinded manner. The second experimental set included the following groups: sham, reflux, reflux + pepstatin and reflux + omeprazole. The same analyses described above were performed, in addition to histological evaluation using Haematoxylin and Eosin staining and cytokine quantification. All histological, biochemical and functional analyses were conducted in a blinded manner.

**TABLE 1:** Distribution of animals by group for experimental set 1.

Experimental group	Initial animals (*n* = 56)	Surviving animals (*n* = 45)	Downstream assays/analyses (*n* = per assay)
Sham	*n *= 6	*n *= 6	Oesophageal and lung wet weight (*n *= 6) Oesophageal and lung myeloperoxidase (*n *= 6)
	*n *= 6	*n *= 6	Expiratory flow (*n *= 6) Tracheal contraction (*n *= 6)
Reflux day 7	*n *= 7	*n *= 6	Oesophageal and lung wet weight (*n *= 6) Oesophageal and lung myeloperoxidase (*n *= 6)
	*n *= 7	*n *= 6	Expiratory flow (*n *= 6) Tracheal contraction (*n *= 6)
Reflux day 21	*n *= 7	*n *= 5	Oesophageal and lung wet weight (*n *= 5) Oesophageal and lung myeloperoxidase (*n *= 6)
	*n *= 7	*n *= 6	Expiratory flow (*n *= 6) Tracheal contraction (*n *= 6)
Reflux day 28	*n *= 8	*n *= 5	Oesophageal and lung wet weight (*n *= 5) Oesophageal and lung myeloperoxidase (*n *= 5)
	*n = 8*	*n *= 5	Expiratory flow (*n *= 5) Tracheal contraction (*n *= 5)

**TABLE 2:** Distribution of animals by group for experimental set 2.

Experimental group	Initial animals (*n* = 76)	Surviving animals (*n* = 61)	Downstream assays/analyses (*n* = per assay)
Sham	*n *= 9	*n *= 9	Oesophageal wet weight (*n *= 6) Oesophageal myeloperoxidase (*n *= 5) Lung myeloperoxidase (*n *= 8) Lung wet weight (*n *= 9) Lung histology (*n *= 5) Expiratory flow (*n *= 8) Expiratory volume minute (*n *= 8)
	*n *= 6	*n *= 6	Interleukin‐6 (*n *= 6) Keratinocyte chemoattractant (*n *= 5) Interleukin‐10 (*n *= 5) Tracheal contraction (*n i*
Reflux day 28	*n *= 10	*n *= 7	Oesophageal wet weight (*n *= 7) Oesophageal myeloperoxidase (*n *= 6) Lung wet weight (*n *= 5) Lung histology (*n *= 6) Expiratory flow (*n *= 5) Expiratory volume minute (*n *= 7)
	*n *= 12	*n *= 9	Lung myeloperoxidase (*n *= 9) Cytokines: IL‐6 (*n *= 8) Keratinocyte chemoattractant (*n *= 8) Interleukin‐10 (*n *= 7) Tracheal contraction (*n *= 6)
Reflux + pepstatin	*n *= 9	*n *= 7	Oesophageal wet weight (*n *= 7) Oesophageal myeloperoxidase (*n *= 6) Lung wet weight (*n *= 6) Lung histology (*n *= 6) Expiratory flow (*n *= 5) Expiratory volume minute (*n *= 6)
	*n *= 12	*n *= 10	Lung myeloperoxidase (*n *= 6) Interleukin‐6 (*n *= 8) Keratinocyte chemoattractant (*n *= 10) Interleukin‐10 (*n *= 9) Tracheal contraction (*n *= 6)
Reflux + omeprazole	*n *= 10	*n *= 7	Oesophageal wet weight (*n *= 6) Oesophageal myeloperoxidase (*n *= 6) Lung wet weight (*n *= 6) Lung histology (*n *= 5) Expiratory flow (*n *= 5) Expiratory volume minute (*n *= 7)
	*n *= 8	*n *= 6	Lung myeloperoxidase (*n *= 6) Interleukin‐6 (*n *= 5) Keratinocyte chemoattractant (*n *= 5) Interleukin‐10 (*n *= 5) Tracheal contraction (*n *= 6)

### 2.4 Treatment

After standardizing the experimental model, two substances were administered: (1) pepstatin (diluted in 4% DMSO), which is a pepsin inhibitor (Sigma^®^, St. Louis, MO, USA), administered at a dose of 0.3 mg/kg via gavage (Nagahama et al., 2006); and (2) omeprazole (diluted in distilled water), which is a PPI (Cristália^®^, Fortaleza, Brazil), administered at a dose of 40 mg/kg intraperitoneally (Silva et al., 2017). The test substances were administered once daily for 28 days.

### 2.5 Evaluation of wet weight

The wet weight of the oesophagus and/or lung was used as an additional indicator of oedema. Organs were dissected, washed with sterile saline, weighed and measured. The results for the oesophagus were expressed in milligrams per centimetre (Silva et al., 2017), and those for the lungs were expressed as centigrams of lung per centigram of body weight (centigrams per centigram) (Júnior et al., 2020).

### 2.6 Assessment of MPO activity

Tissue samples from the oesophagus and lungs were homogenized in potassium phosphate buffer containing 0.5% hexadecyltrimethylammonium bromide (pH 6.0). Thereafter, the homogenate was centrifuged at 13,416 × g for 5 min at 4°C, and 10 µL of the supernatant was used for analysis. A reading solution (distilled water, *o*‐dianisidine and 5% hydrogen peroxide) was prepared. During the assay, as hydrogen peroxide was degraded, superoxide anion production occurred, which was responsible for the conversion of *o*‐dianisidine. The results were expressed in units (U) of MPO per milligram of tissue (Bradley et al., 1982).

### 2.7 Cytokine measurement (interleukin‐6, keratinocyte chemoattractant and interleukin‐10)

The lung cytokine levels were also measured. These fragments were added to protease inhibitor buffer (500 µL of buffer for every 100 mg of tissue), processed in a tissue homogenizer, and centrifuged at 3000 rpm at 4°C for 10 min. The MILLIPLEX^®^ Cytokine/Chemokine 4‐plex Kit (Millipore Corporation, Billerica, MA, USA) with interleukin (IL)‐6, keratinocyte chemoattractant (KC) and IL‐10 detection antibodies for mice was chosen. In summary, lung tissue homogenate samples were diluted using a ratio of 1:2 in assay buffer, and 25 µL of standard solutions, quality control solutions and duplicate samples were added to the plate. This procedure was followed by the addition of 25 µL of magnetic beads. The plate was sealed, covered with aluminum foil, and incubated overnight on a plate shaker at 4°C. The plate was washed three times, and 25 µL of detection antibody was added to each well. After incubating the plate at room temperature for 1 h, 25 µL of streptavidin–phycoerythrin was added to each well. The plates were then sealed, covered, and incubated for 30 min at room temperature. The plate went through a final series of washes before the addition of 150 µL of driving fluid. Marker concentrations were measured on a Luminex^®^ MAGPIX^®^ with xPONENT^®^ software (Luminex Corporation, Austin, TX, USA) (Richens et al., 2010).

### 2.8 Histopathological investigation

Lung samples were removed and fixed in 10% buffered formalin. They were subsequently embedded in paraffin using standard methods, cut into 5‐µm‐thick sections with the aid of a microtome, and stained with Haematoxylin and Eosin to assess alveolitis. The changes evaluated in the lungs included the absence of alveolitis (score 0), mild alveolitis (thickening of the alveolar septum by infiltration and injury to ≥20% of the lung; score 1), moderate alveolitis (20%–50% of the lung; score 2) and severe alveolitis (>50% of the lung; score 3) (Liu et al., 2020).

### 2.9 Pulmonary spirometry

The mice were anaesthetized (tribromoethanol 100 mg/kg, Sigma‐Aldrich, St. Louis, MO, USA) intraperitoneally, tracheostomized, and placed in a spirometer for small animals (AD Instruments^®^, Sydney, NSW, Australia), where they remained for 10 min. Expiratory flow and minute volume were evaluated, and data were obtained and analysed using LabChart^®^ software (Guenther et al., 2010).

### 2.10 Contractility evaluation of trachea

Tracheal strips were mounted vertically in an organ bath containing Krebs solution (37°C at pH 7.4) composed of (mM): 118.4 NaCl, 4.7 KCl, 1.2 MgSO_4_, 2.5 CaCl_2_, 2.5 NaHCO_3_, 11.7 glucose and 3.2 KH_2_PO_4_. The organ bath was continuously oxygenated with 95% O_2_ and 5% CO_2_. Thereafter, a basal tension of 500 mg was applied to the tissues, and a stabilization period of 50 min was allocated for adaptation to the new conditions, with the Krebs solution replaced by a new one every 15 min. Evaluation of the contractile behaviour of the tracheal segments was performed using concentration–effect curves with KCl (10–160 mM) or with the muscarinic agonist carbachol (CCh; 0.01–100 µM) (Gadelha et al., 2019).

### 2.11 Statistical analysis

Statistical analysis was performed using the Shapiro–Wilk normality test. Subsequently, an ANOVA was performed, followed by Dunnett's multiple‐comparison test. For comparisons involving sham and reflux groups, the sham group was defined as the reference control group. In experiments evaluating treatment interventions within reflux group animals, the reflux group was defined as the reference control group. The Kruskal–Wallis non‐parametric test was used to analyse histological changes, followed by Dunn's multiple‐comparison test. A two‐way repeated‐measures ANOVA followed by Dunnett's multiple‐comparison test was used to analyse the concentration–response curves, with the appropriate reference group specified in the corresponding analyses. The results are expressed as the mean ± SD or as the median with minimum and maximum. For parametric comparisons, the mean difference (MD) and its corresponding 95% confidence interval (95% CI) were also calculated and reported. Differences were considered statistically significant at *P* < 0.05, and the data were analysed using GraphPad Prism 7.0.

## 3 RESULTS

### 3.1 Temporal progression of oesophageal and pulmonary changes in a murine reflux model

Table 3 shows a significant increase in oesophageal wet weight and MPO activity in the reflux group on day 7 compared with those in the sham group, with these alterations persisting until day 28. In the lungs, wet weight increased significantly from day 21 onwards and remained elevated on day 28. Pulmonary MPO activity showed a progressive and significant increase, reaching its highest level on day 28. Respiratory function parameters revealed a progressive and significant reduction in expiratory flow from day 21 onwards. Additionally, tracheal contractility increased significantly only on day 28 in response to both KCl (80 mM) and carbachol (1 µM). Therefore, day 28 was selected as the ideal time point for standardizing the experimental model, because of the higher levels of inflammation and decreased functional responses observed in both spirometry and tracheal reactivity.

**TABLE 3:** Time course of pulmonary morphofunctional changes secondary to gastro‐oesophageal reflux in an experimental murine model, assessed on days 7, 21 and 28 after reflux model induction.

Parameter	Sham	Reflux day 7	Reflux day 21	Reflux day 28	*P*‐value
Oesophageal wet weight, mg/cm	9.4 ± 0.9	19.7 ± 2.2	19.2 ± 5.8	15.2 ± 3.6	0.0001
Oesophageal myeloperoxidase, U/mg of tissue	0.8 ± 0.711	18.6 ± 4.865	11.1 ± 3.848	7.3 ± 2.793	0.001
Lung wet weight, cg/cg	0.44 ± 0.0362	0.50 ± 0.0263	0.53 ± 0.0644	0.54 ± 0.0291	0.001
Lung myeloperoxidase, U/mg of tissue	2.2 ± 1.020	5.2 ± 2.262	11.1 ± 4.393	20.0 ± 4.531	0.0001
Expiratory flow, mL/s	0.025 ± 0.0038	0.023 ± 0.0037	0.019 ± 0.0041	0.018 ± 0.0017	0.001
Minute volume, mL/min	139.6 ± 5.179	155.7 ± 7.128	120.1 ± 14.62	122.5 ± 15.00	0.0002
Contraction (80 mM KCl), mg/mg of tissue	0.53 ± 0.067	0.63 ± 0.102	0.60 ± 0.140	0.79 ± 0.153	0.0001
Contraction (1 µM CCh), mg/mg of tissue	0.57 ± 0.110	0.70 ± 0.068	0.62 ± 0.105	0.84 ± 0.206	0.0001

*Note*: Data are expressed as the mean ± SD. Exact *P*‐values for all comparisons are shown. Statistical analysis was performed using the Shapiro–Wilk test, followed by one‐way ANOVA and Dunnett's test. *P* < 0.05 vs. Sham. Number of animals per group, *n* = 5 or 6.

### 3.2 Pepstatin and omeprazole decrease oesophageal inflammation in a murine reflux model

As previously observed, the reflux group (*n* = 7; 16.2 ± 3.2 mg/cm) developed oesophageal oedema (MD = 5.78, 95% CI: 3.84 to 7.71; *P* = 0.0001) compared with the sham group (*n* = 6; 9.4 ± 0.9 mg/cm). Treatment with pepstatin (*n* = 7; 9.9 ± 1.3 mg/cm) significantly reduced (MD = 5.23, 95% CI: 3.37 to 7.08; *P *= 0.0001) oesophageal inflammation compared with the reflux group. Likewise, treatment with omeprazole (*n* = 6; 12.3 ± 1.8 mg/cm) also significantly reduced (MD = 2.85, 95% CI: 0.92 to 4.79; *P* = 0.0032) oesophageal inflammation compared with the reflux group; (Figure 1a).

**FIGURE 1:**
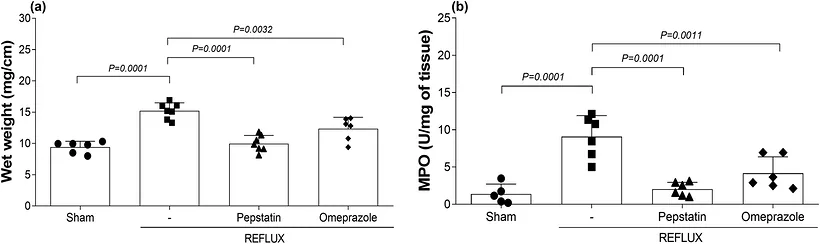
Pepstatin and omeprazole mitigate oesophageal inflammation in a murine reflux model: (a) oesophageal wet weight; and (b) myeloperoxidase (MPO) activity. Measurements were performed in the sham, reflux, reflux + pepstatin and reflux + omeprazole groups at 28 days postoperatively. Data are shown as the mean + SD. Exact *P*‐values for all comparisons are shown in the figure. Statistical analysis was performed using the Shapiro–Wilk test, followed by one‐way ANOVA and Dunnett's test. *P* < 0.05 vs. reflux. *n* = 5–7 mice per group.

Regarding MPO activity, the reflux group (*n* = 6; 9 ± 2.8 U/mg of tissue) showed significantly higher levels (MD = 7.68, 95% CI: 4.61 to 10.76; *P* = 0.0001) than the sham group (*n* = 5; 1.3 ± 1.3 U/mg of tissue). Reflux + pepstatin group (*n* = 6; 2 ± 0.9 U/mg of tissue) showed significantly reduced inflammation (MD = 7.05, 95% CI: 4.12 to 9.98; *P *= 0.0001) compared with the reflux group. Likewise, the reflux + omeprazole group (*n* = 6; 4.1 ± 2.1 U/mg of tissue) also showed significantly reduced inflammation (MD = 4.90, 95% CI: 1.97 to 7.83; *P* = 0.0011) compared with the reflux group (Figure 1b).

### 3.3 Pepstatin decreases pulmonary inflammatory parameters in a murine reflux model

Regarding pulmonary oedema, the reflux group (*n* = 5; 0.54 ± 0.029 cg/cg) showed a significant increase (MD = 0.09, 95% CI: 0.03 to 0.14; *P *= 0.0016) compared with the sham group (*n* = 9; 0.44 ± 0.036 cg/cg). The administration of pepstatin (*n* = 6; 0.45 ± 0.037 cg/cg; vs. reflux group) significantly reduced wet weight (MD = 0.08, 95% CI: 0.02 to 0.14; *P *= 0.0051). However, omeprazole (*n* = 6; 0.48 ± 0.055 cg/cg; vs. reflux group) did not show a significant difference (MD = 0.05, 95% CI: −0.008 to 0.11; *P* = 0.09; Figure 2a).

**FIGURE 2:**
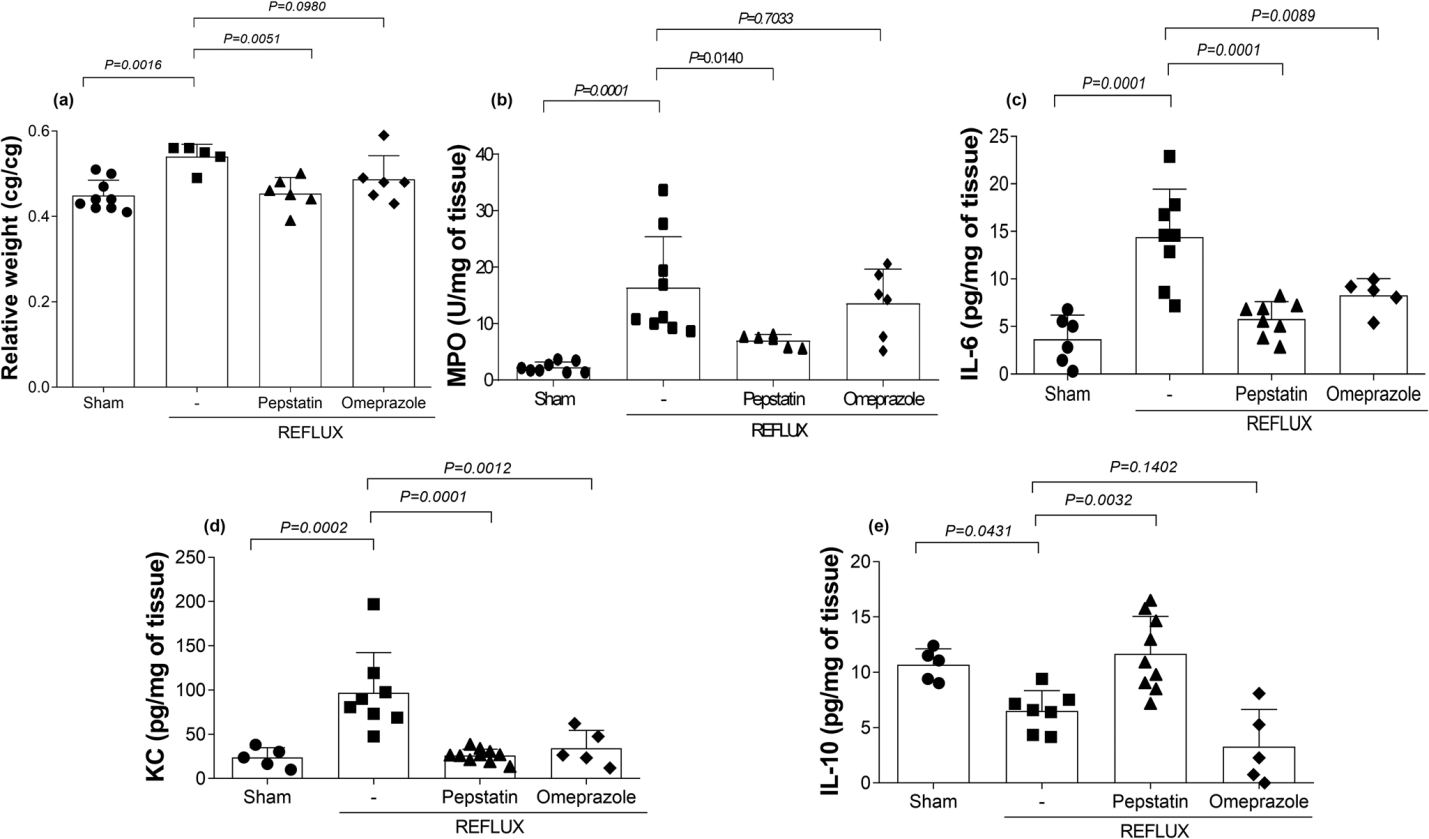
Effect of pepstatin and omeprazole on pulmonary inflammation in a murine reflux model: (a) pulmonary wet weight; (b) myeloperoxidase (MPO) activity; (c) keratinocyte chemoattractant (KC); (d) interleukin (IL)‐6; and (e) IL‐10. Measurements were performed in the sham, reflux, reflux + pepstatin and reflux + omeprazole groups 28 days postoperatively. Data are expressed as the mean + SD. Exact *P*‐values for all comparisons are shown in the figure. Statistical analysis was performed using the Shapiro–Wilk test, followed by one‐way ANOVA and Dunnett's test. *P* < 0.05 vs. reflux. *n* = 5–10 mice per group.

The reflux group (*n* = 9; 16.4 ± 8.9 U/mg of tissue) exhibited a significant increase (MD = 14.13 ‐ 95% CI: 7.04 to 21.22; *P* = 0.0001) in MPO activity compared with the sham group (*n* = 8; 2.2 ± 0.9 U/mg of tissue). MPO activity significantly decreased (MD = 9.40, 95% CI: 1.71 to 17.10; *P *= 0.014) in the reflux + pepstatin group (*n* = 6; 6.9 ± 1.0 U/mg of tissue; vs. reflux group), whereas the use of omeprazole (*n* = 6; 13.5 ± 6.0 U/mg of tissue; vs. reflux group) did not show a significant difference in MPO activity (MD = 2.80, 95% CI: −4.89 to 10.49; *P *= 0.70; Figure 2b).

IL‐6 levels (Figure 2c) were significantly increased (MD = 10.75, 95% CI: 6.29 to 15.21; *P *= 0.0001) in the reflux group (*n* = 8; 14.4 ± 5.0 pg/mg of protein) compared with the sham group (*n* = 6; 3.6 ± 2.5 pg/mg of protein). Treatment with pepstatin significantly reduced (MD = 8.63, 95% CI: 4.50 to 12.76; *P *= 0.0001) IL‐6 levels in the reflux + pepstatin group (*n* = 8; 5.7 ± 1.8 pg/mg of protein; vs. reflux group). Likewise, omeprazole treatment also reduced (MD = 6.13, 95% CI: 1.42 to 10.84; *P *= 0.0089) IL‐6 levels in the reflux + omeprazole group (*n* = 5; 8.2 ± 0.7 pg/mg of protein; vs. reflux group).

KC levels (Figure 2d) followed a similar pattern, showing a significant increase (MD = 73.03, 95% CI: 34.60 to 111.15; *P *= 0.0002) in the reflux group (*n* = 8; 96.8 ± 45.6 pg/mg of protein) compared with the sham group (*n* = 7; 23.8 ± 11.1 pg/mg of protein; *P *= 0.0001). Pepstatin treatment significantly reduced (MD = 70.86, 95% CI: 38.88 to 102.8; *P *= 0.0001) KC levels (*n* = 10; 25.9 ± 7.2 pg/mg of protein; vs. reflux group), and omeprazole also decreased (MD = 62.49, 95% CI: 24.06 to 100.9; *P *= 0.0012) KC levels (*n* = 5; 39.9 ± 18.3 pg/mg of protein; vs. reflux group).

IL‐10 levels (Figure 2e) were significantly decreased (MD = −4.17, 95% CI: −8.23 to −0.11; *P *= 0.0043) in the reflux group (*n* = 7; 6.5 ± 1.8 pg/mg of protein) compared with the sham group (*n* = 5; 10.6 ± 1.4 pg/mg of protein). Pepstatin treatment preserved IL‐10 levels in the reflux + pepstatin group (*n* = 9; 11.6 ± 3.3 pg/mg of protein; MD = −5.16, 95% CI: −8.66 to −0.16; *P *= 0.0032 vs. reflux group). In contrast, IL‐10 levels in the reflux + omeprazole group (*n* = 5; 3.2 ± 3.3 pg/mg of protein) were not significantly different compared with the reflux group (MD = 3.23, 95% CI: −0.83 to 7.29; *P *= 0.14).

### 3.4 Pepstatin attenuates histopathological alterations in the lungs in a murine reflux model

The histopathological evaluation scores (Figure 3) showed that the reflux group [*n* = 6; 2 (2–3)] had a significantly higher percentage of alveolitis (*P *= 0.0040) than the sham group [*n* = 5; 1 (0–1)]. Treatment with pepstatin [*n* = 6; 1.5 (1–2)] significantly reduced the incidence of alveolitis (*P *= 0.024), whereas omeprazole [*n* = 5; 3 (2–3)] did not show a significant difference.

**FIGURE 3:**
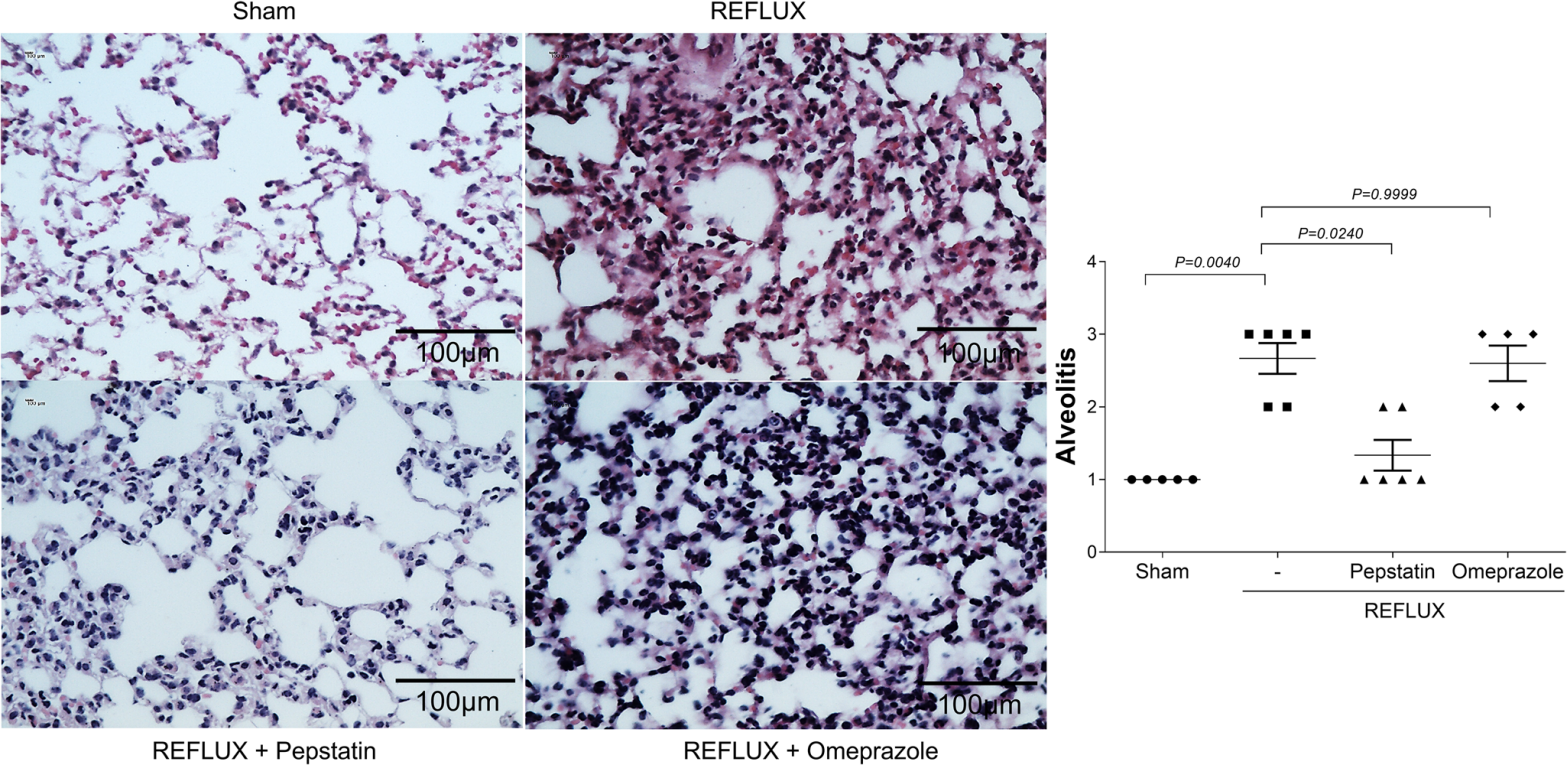
Histological scores and photomicrographs of the lungs of mice within a murine reflux model treated with pepstatin and omeprazole. Left, photomicrographs of alveolitis in the lungs of the sham, reflux, reflux + pepstatin, and reflux + omeprazole groups (scale bar = 100 µm). Right, histological scores. Data are expressed as medians. Exact *P*‐values for all comparisons are shown in the figure. Statistical analysis was performed using the Kruskal–Wallis test, followed by Dunn's test. *P* < 0.05 vs. reflux. *n* = 5–6 mice per group.

### 3.5 Pepstatin restores pulmonary function in a murine reflux model

In the reflux group (*n* = 5; 0.012 ± 0.001 mL/s), expiratory flow was significantly reduced (MD = −0.008, 95% CI: −0.010 to −0.005; *P *= 0.0001) compared with the sham group (*n* = 8; 0.02 ± 0.001 mL/s). Upon administration of pepstatin (*n* = 5; 0.016 ± 0.001 mL/s; vs. reflux group), a significant improvement in this functional parameter was observed (MD = −0.003, 95% CI: −0.006 to −0.0008; *P *= 0.0101), whereas omeprazole (*n* = 5; 0.014 ± 0.002 mL/s; vs. reflux group) did not result in a significant difference (MD = −0.002, 95% CI: −0.004 to −0.0006; *P *= 0.164; Figure 4a). In terms of minute volume, the reflux group (*n* = 8; 123.0 ± 2.6 mL) presented significantly lower values (MD = −11.57, 95% CI: −18.33 to −4.82; *P *= 0.0007) than the sham group (*n* = 8; 134.5 ± 8.1 mL; Figure 4b). Treatment with pepstatin (*n* = 6; 131.3 ± 3.7 mL; vs. reflux group) significantly improved this parameter (MD = −8.38, 95% CI: −15.68 to −1.08; *P *= 0.0101), whereas omeprazole (*n* = 7; 129.6 ± 4.8 mL; vs. reflux group) did not show a significant difference (MD = −6.59, 95% CI: −13.59 to 0.39; *P *= 0.067).

**FIGURE 4:**
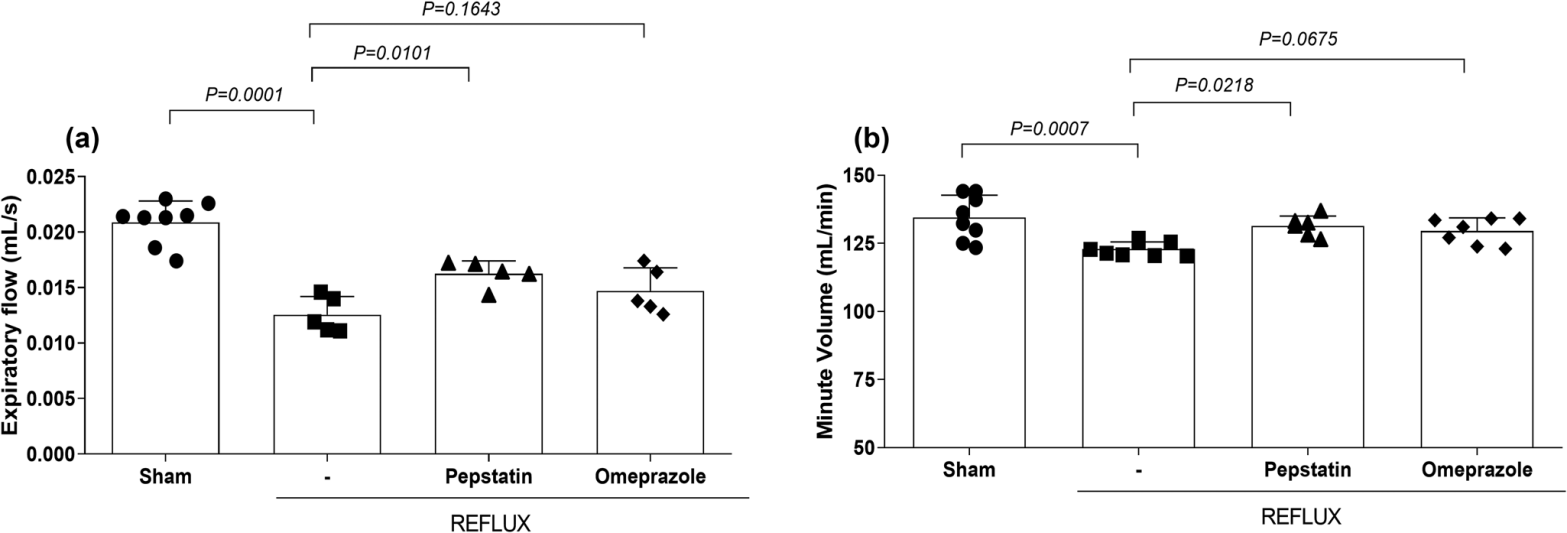
Protective effects of pepstatin and omeprazole on pulmonary function in a murine reflux model: (a) expiratory flow; and (b) minute volume. Measurements were performed in the sham, reflux, reflux + pepstatin and reflux + omeprazole groups 28 days postoperatively. Data are shown as the mean + SD. Exact *P*‐values for all comparisons are shown in the figure. Statistical analysis was performed using the Shapiro–Wilk test, followed by one‐way ANOVA and Dunnett's test. *P* < 0.05 vs. reflux. *n* = 5–8 mice per group.

The concentration–response curve for KCl‐induced tracheal contraction (Figure 5a) revealed significant effects of KCl concentration [*F*(1.352, 35.15) = 94.83, *P *= 0.0001], experimental group [*F*(3, 26) = 2.995, *P *= 0.0490] and the interaction between these factors [*F*(27, 234) = 5.569, *P *= 0.0001]. At KCl concentrations ranging from 80 to 160 mM, the reflux group (*n* = 6; 0.7919 ± 0.1534 at 80 mM and 0.6970 ± 0.0960 at 160 mM) exhibited significantly greater tracheal reactivity than the sham group (*n* = 6; 0.5311 ± 0.0677 at 80 mM and 0.4800 ± 0.0592 at 160 mM; 80 mM, MD = 0.2608, 95% CI: 0.025 to 0.49, *P *= 0.0337; 160 mM, MD = 0.2170, 95% CI: 0.14 to 0.28, *P *= 0.0002). Reflux + pepstatin (*n* = 6; 0.6299 ± 0.1232 at 100 mM and 0.5329 ± 0.7984 at 160 mM) significantly reduced tracheal hyperreactivity compared with the reflux group (0.8585 ± 0.1321 at 100 mM and 0.6970 ± 0.0960 at 160 mM) at KCl concentrations ranging from 100 to 160 mM (100 mM, MD = 0.2285, 95% CI: 0.049 to 0.40, *P *= 0.0149; 160 mM, MD = 0.1642, 95% CI: 0.072 to 0.25, *P *= 0.0014). In contrast, the reflux + omeprazole group (*n* = 6; 0.4095 ± 0.08220 at 10 mM and 0.6377 ± 0.0995 at 160 mM) showed no significant differences at any KCl concentration compared with the reflux group (0.4181 ± 0.09774 at 10 mM and 0.6970 ± 0.09604 at 160 mM; 10 mM, MD = 0.006150, 95% CI: −0.13 to 0.15, *P *= 0.9982; 160 mM, MD = 0.05929, 95% CI: −0.030 to 0.14, *P *= 0.2237).

**FIGURE 5:**
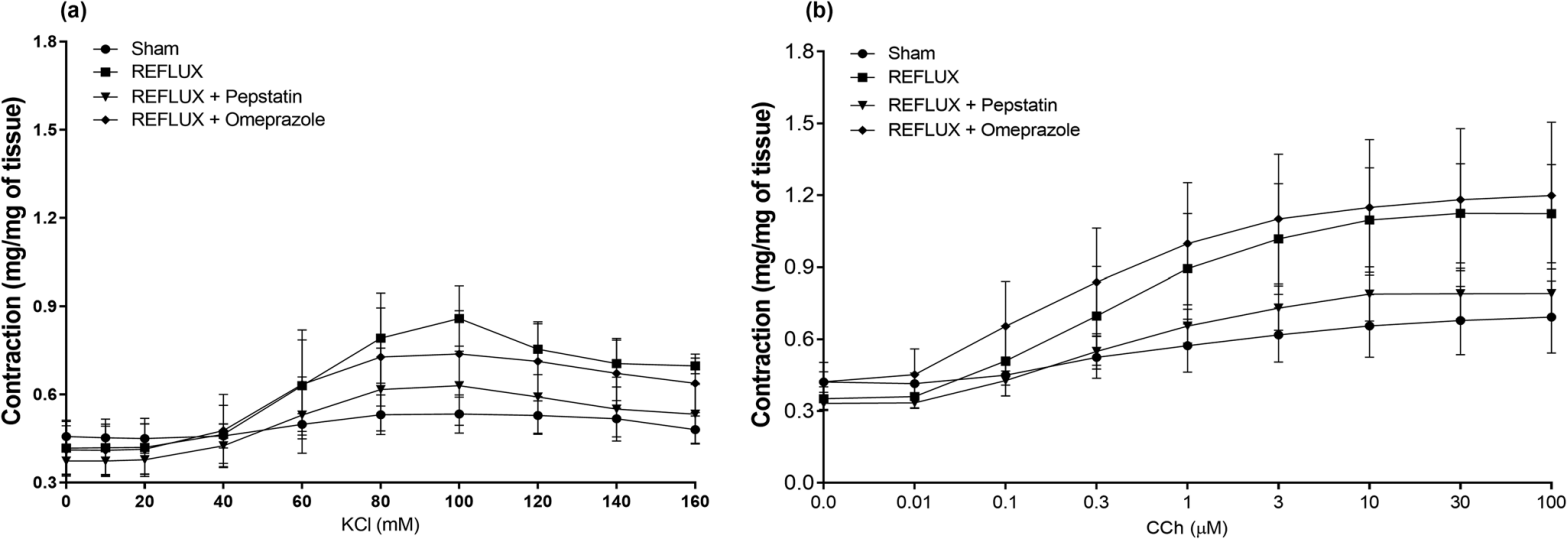
Modulation of tracheal contractile responses by pepstatin and omeprazole in a murine reflux model. Concentration–response curves for KCl (1–160 mM) (a) and the muscarinic agonist carbachol (CCh; 0.01–100 µM) (b). Measurements were performed in the sham, reflux, reflux + omeprazole and reflux + pepstatin groups at 28 days postoperatively. Data are expressed as the mean ± SD. Exact *P*‐values for all comparisons are shown in the figure. Statistical analysis was performed using a two‐way repeated‐measures ANOVA, followed by Dunnett's test. *P* < 0.05 vs. reflux. *n* = 6 mice per group.

Evaluation of tracheal reactivity to the muscarinic agonist carbachol (CCh; Figure 5b) revealed significant effects of CCh concentration [*F*(1.559, 38.98) = 212.3, *P *= 0.0001], treatment group [*F*(3, 25) = 8.467, *P *= 0.0005] and the interaction between these factors [*F*(24, 200) = 10.55, *P *= 0.0001]. At CCh concentrations ranging from 3 to 100 µM, the reflux group (*n* = 6; 1.018 ± 0.2046 at 3 µM and 1.123 ± 0.2019 at 100 µM) exhibited significantly greater tracheal reactivity than the sham group (*n* = 6; 0.6183 ± 0.1133 at 3 µM and 0.6928 ± 0.1501 at 100 µM; 3 µM, MD = 0.3994, 95% CI: 0.048 to 0.75, *P *= 0.0309; 100 µM, MD = 0.4305, 95% CI: 0.11 to 0.74, *P *= 0.0122). Reflux + pepstatin (*n* = 6; 0.7901 ± 0.1599 at 30 µM and 0.7910 ± 0.1633 at 100 µM) significantly attenuated tracheal hyper‐reactivity compared with the reflux group (1.124 ± 0.1990 at 30 µM and 1.123 ± 0.2019 at 100 µM) at CCh concentrations of 30 and 100 µM (30 µM, MD = 0.3341, 95% CI: 0.017 to 0.65, *P *= 0.0408; 100 µM, MD = 0.3323, 95% CI: 0.019 to 0.64, *P *= 0.0401). Conversely, the reflux + omeprazole group (*n* = 6; 0.4528 ± 0,1187 at 0.01 µM and 1.199 ± 0.3471 at 100 µM) showed no significant differences at any CCh concentration compared with the reflux group (0.3606 ± 0.0355 at 0.01 µM and 1.123 ± 0.2019 at 100 µM; 0.01 µM, MD = −0.09219, 95% CI: −0.19 to 0.014, *P *= 0.0941; 100 µM, MD = 0.07560, 95% CI: −0.43 to 0.27, *P *= 0.8790).

## 4 DISCUSSION

In clinical practice, patients with GORD‐associated pulmonary disorders can present with asthma, pulmonary fibrosis, chronic cough and bronchitis (Durazzo et al., 2020). Despite these well‐recognized clinical associations, important gaps remain in understanding the pathophysiological mechanisms underlying the development of these extra‐oesophageal manifestations, particularly the molecular pathways involved. Furthermore, current therapeutic options remain limited, because no pharmacological intervention has been shown to prevent or treat GORD‐associated pulmonary complications effectively, resulting in a substantial impact on patients’ quality of life (Drug et al., 2022). In this context, the development of experimental models is essential to investigate the mechanisms underlying reflux‐induced extra‐oesophageal disorders and provides a valuable platform for both mechanistic studies and the preclinical evaluation of new therapeutic strategies.

Accordingly, the present study was conducted using the murine reflux model established by Silva et al. (2017). This model was chosen because it has already demonstrated inflammation and loss of mucosal integrity in both the oesophagus (Nicolau et al., 2019; Silva et al., 2017) and the larynx (Figueiredo et al., 2020; Sales et al., 2023, 2024). Additionally, the fact that mice are quadrupeds and remain in a horizontal position for most of the time might facilitate reflux induced by the model, thereby contributing to an increase in microaspiration events. In this context, our data showed that oesophageal inflammation persisted, albeit with a slight reduction, by day 28. The wet weight of the lungs increased significantly. Pulmonary MPO activity showed a progressive and significant increase over time, indicating a continuous inflammatory response. The data revealed an increase in IL‐6 and KC levels, reduction in IL‐10 levels, and alveolitis. Respiratory function parameters revealed a progressive and significant reduction in expiratory flow. Moreover, tracheal contractility significantly increased in response to KCl and CCh. These results suggest that this experimental model of gastro‐oesophageal reflux induces progressive inflammatory and functional alterations in the lungs, with late‐onset pulmonary inflammation associated with impaired respiratory function owing to microaspiration of gastric contents.

The literature has shown that chronic endotracheal instillation of gastric contents leads to infiltration of inflammatory cells, particularly neutrophils and macrophages, and an increase in the expression of pro‐inflammatory cytokines, such as IL‐1β, IL‐6 and tumour necrosis factor‐α in the lungs. This results in structural pulmonary changes, including septal thickening and early fibrosis (Appel et al., 2007). Furthermore, studies have revealed that different gastric components, when applied individually, cause distinct responses in the lung tissue (Samareh Fekri et al., 2014). Pepsin plays a key role in the progression of pulmonary damage. Chen et al. (2019) have shown that chronic gastric content aspiration, particularly when combined with pepsin, induces lung inflammation and fibrosis in rats. Additionally, Hurley et al. (2019) demonstrated that when acid‐injured lung tissue comes into contact with pepsin, there is a significant increase in neutrophil migration through the damaged epithelium.

In this context, it is hypothesized that targeting pepsin activity might play a relevant role in respiratory diseases associated with gastro‐oesophageal reflux because of its enzymatic capacity to remain active at pH levels 6.0 and its structural stability up to pH 8.0. These characteristics allow the enzyme to be reactivated during subsequent episodes of acid reflux (Piper & Fenton, 1965) or through cellular endocytosis, where it regains its activity within the acidified intracellular compartments (Samuels & Johnston, 2020). Although PPIs are effective in increasing the gastric pH and, consequently, reducing the conversion of pepsinogen to pepsin, they do not directly affect the frequency of reflux episodes (Rettura et al., 2021). In this scenario, the previously inactivated yet structurally stable pepsin can be reactivated and exert cytotoxic effects, contributing to tissue injury independent of acid suppression. This phenomenon might partly explain the limited therapeutic efficacy of omeprazole observed in this study.

Given the harmful effects attributed to pepsin and the limitations of current acid‐suppressive approaches, pharmacological inhibition of pepsin has emerged as a potential strategy for investigating reflux‐associated tissue injury. In this context, we investigated pepstatin A (a hexapeptide with the sequence isovaleryl‐l‐valyl‐l‐valylstatylalanil‐statine) derived from *Streptomyces*. Pepstatin A is a potent inhibitor of almost all aspartic proteases, including those from the human immunodeficiency virus (HIV), pepsin, renin, cathepsin D, chymosin, *Aspergillus niger* protease B and other microbial acid proteases, and it does not inhibit serine, cysteine or metalloproteinases (Purushothaman et al., 2021).

Our data demonstrated that pepstatin administration attenuated pulmonary inflammation. We hypothesized that, by inhibiting pepsin enzymatic activity, microaspirated gastric contents lose an important stimulus for activating the innate immune response, reducing the activation of alveolar epithelial cells and resident macrophages. As a consequence, there is less production of IL‐6 and KC, resulting in reduced neutrophil recruitment to the lung. IL‐6 acts as an important amplifier of the innate immune response, promoting leucocyte activation and sustaining the inflammatory cascade (Hunter, Jones, 2015), whereas KC is the main chemokine responsible for neutrophil recruitment to the lung (Sawant et al., 2016). In contrast, IL‐10 is not only an anti‐inflammatory cytokine but also an important regulator of inflammation resolution, limiting macrophage activation and exacerbated neutrophilic inflammation, while preserving lung tissue integrity (Neumann et al., 2019). Thus, the simultaneous reduction of IL‐6 and KC, together with the preservation of IL‐10 levels, might be consistent with the hypothesis that pepsin inhibition is involved in regulation of the pulmonary inflammatory response, potentially attenuating pathways related to neutrophil recruitment and innate immune amplification, while preserving mechanisms involved in the resolution of inflammation.

Finally, our findings demonstrated that a reduction in inflammation had beneficial effects on pulmonary function, as evidenced by improvements in expiratory flow, minute volume and tracheal contractility through both electromechanical and pharmacomechanical mechanisms. These findings are consistent with those reported in the literature. Studies have demonstrated that pepsin inhibition might be an attractive therapeutic approach for patients with oesophageal or extra‐oesophageal disorders associated with GORD. Nagahama et al. (2006) showed that intragastric administration of pepstatin in a rat model of erosive oesophagitis significantly prevented oesophageal injury without affecting basal acid secretion. Kim et al. (2018) conducted in vitro studies of tonsillar hypertrophy using patient biopsies to investigate the role of pepsin in tissue damage. They found increased numbers of CD4^+^ cells and elevated levels of IL‐2 and interferon‐γ following pepsin exposure, all of which were significantly reduced by pepstatin treatment. Hurley et al. (2019) demonstrated that the addition of pepstatin A significantly inhibits both neutrophil migration and the loss of pulmonary epithelial barrier integrity induced by pepsin at pH 3 in an in vitro model.

Based on our findings, we hypothesized that pepstatin begins its inhibition after the conversion of pepsinogen to pepsin (Roberts & Taylor, 2003). Consequently, pepstatin binding induces structural changes in pepsin, creating an active site close to the inhibitor (Fujinaga et al., 1995). In this case, administration via gavage was appropriate, because the inhibitory efficacy of pepstatin is maintained within a pH range of 1.5–3.8, thus decreasing its effect at higher pH levels (Roberts & Taylor, 2003). Therefore, it is believed that when gastric contents are refluxed, the deleterious effects of pepsin on airway epithelial cells are reduced. Although our findings are consistent with inhibition of pepsin before reflux and microaspiration, an additional mechanism cannot be excluded. Because pepsin remains structurally stable at near‐neutral pH and can be reactivated after subsequent acidification or within acidified intracellular compartments following endocytosis (Johnston et al., 2007; Samuels & Johnston, 2020), pepstatin that reaches the airway lumen concurrently with aspirated gastric contents could, theoretically, inhibit pepsin locally. This possibility is supported by in vitro evidence showing that pepstatin prevents pepsin‐induced epithelial injury and neutrophil migration in pulmonary epithelial models (Hurley et al., 2019). However, because pepstatin was administered orally by gavage and exhibits low systemic absorption and limited bioavailability (Marciniszyn et al., 1976; Umezawa et al., 1970), our experimental design does not allow us to distinguish gastric pre‐aspiration inhibition from direct airway inhibition in vivo. Beyond these experimental limitations, the translation of pepstatin to clinical applications faces severe hurdles. Although pepstatin is a potent inhibitor of pepsin, its clinical utility is restricted by its poor water solubility, owing to its highly hydrophobic structure, which is rich in non‐polar residues, such as statin. The absence of ionizable groups prevents adequate aqueous solubilization, necessitating the use of organic solvents, such as methanol or DMSO, for dissolution. Furthermore, its unfavourable pharmacokinetic profile (characterized by low absorption and poor bioavailability) significantly compromises its therapeutic potential (Marciniszyn et al., 1976; Umezawa et al., 1970).

Therefore, studies focusing on aspartic protease inhibitors have attracted increasing attention. Johnston et al. (2023) reported that darunavir, an antiretroviral drug approved by the US Food and Drug Administration for HIV/acquired immunodeficiency syndrome, binds to and inhibits pepsin, thus effectively preventing pepsin‐induced laryngeal inflammation and mucosal injury in a murine laryngopharyngeal reflux model. Samuels et al. (2023) and Blaine‐Sauer et al. (2023) demonstrated that aspartic protease inhibitors attenuated the disruption of laryngeal and oesophageal epithelial barriers in cell culture models. Likewise, Sales et al. (2024) demonstrated that darunavir reduces laryngeal inflammation and preserves mucosal integrity in a murine model of laryngopharyngeal reflux. In another study, the same authors also showed that pepsin inhibition attenuates oesophageal inflammation in a GORD model (Sales et al., 2026), suggesting a potential protective effect on the inflammatory response and tissue integrity in the context of gastro‐oesophageal reflux. Although the literature has long shown that pepsin acts as an aggressive or potentiating factor in the inflammatory process of reflux‐associated diseases, our study provides new evidence demonstrating that inhibition of this enzyme leads to the attenuation of pulmonary inflammation, resulting in an improved functional response in a murine model of reflux.

Taken together with previous evidence, our findings suggest that pepsin inhibition might be beneficial for patients with: (1) GORD with inadequate response to PPIs and/or those with persistent symptoms despite mucosal oesophageal healing with PPI therapy (Katz et al., 2022; Mermelstein et al., 2018; Mönnikes et al., 2013); (2) patients with aspiration‐related lung diseases, such as recurrent aspiration pneumonia and pulmonary fibrosis (Iov et al., 2022; Reynolds et al. 2023; Savarino et al., 2013); and (3) those undergoing lung transplantation, in whom gastric reflux‐mediated aspiration has been associated with allograft injury and chronic lung allograft dysfunction (Hathorn et al., 2017). However, further studies are needed to confirm these hypotheses.

This study has several limitations. It is unlikely that bile acids and pancreatic enzymes contributed to the pathogenesis of this reflux model, given that partial pyloric stenosis was induced, thus preventing duodenal contents from refluxing into the stomach. To support this assumption, we evaluated a bile salt chelator in this model and observed no significant effects on either oesophageal or pulmonary injury (data not shown). The doses of omeprazole and pepstatin were selected based on previously published studies. A further limitation concerns the translational applicability of pepstatin, which was used purely as a pharmacological tool to demonstrate the role of pepsin in reflux‐associated pulmonary inflammation rather than as a therapeutic candidate. Its clinical utility is restricted by unfavourable physicochemical and pharmacokinetic properties, including high hydrophobicity, poor aqueous solubility and low bioavailability. Nevertheless, the therapeutic potential of pepsin inhibition extends beyond pepstatin. Small‐molecule aspartic protease inhibitors, such as fosamprenavir, have effectively attenuated pepsin‐mediated tissue injury in preclinical models and are currently undergoing phase II clinical evaluation for reflux disease. These programmes offer a viable translational path for targeting pepsin, although the safety, efficacy and ability of pepsin inhibition to prevent lower‐airway inflammation in humans remain to be established. In addition, the surgically induced murine model reproduces important features of reflux‐mediated oesophageal and pulmonary injury but does not fully capture the complexity, chronicity and heterogeneity of human GORD. Differences in anatomy, physiology, immune responses and reflux composition between mice and humans should be considered when interpreting these findings. Moreover, although the experimental period was sufficient to evaluate reflux‐induced inflammation and pulmonary functional impairment, it was unable to develop structural changes, such as airway remodelling and pulmonary fibrosis. Therefore, additional studies using longer follow‐up periods are warranted to determine whether sustained pepsin inhibition can prevent chronic tissue remodelling and fibrotic progression associated with reflux‐induced lung injury.

## 5 CONCLUSION

In conclusion, this study suggests that the reflux model induces progressive pulmonary inflammation and functional impairment. Although omeprazole treatment increased gastric pH, it did not fully prevent lung injury. In contrast, administration of pepstatin mitigated the levels of inflammatory markers and was associated with improved pulmonary function. Therefore, pepsin might play a significant pro‐inflammatory and cytotoxic role in reflux‐associated respiratory diseases.

## AUTHOR CONTRIBUTIONS

All experiments were conducted at the Federal University of Ceará (UFC), Brazil. Thiago Meneses Araújo Leite Sales: conceptualization; methodology; investigation; data curation; formal analysis; visualization; writing—original draft; writing—review & editing validation. Humberto Barbosa da Costa Filho, Kalinne Kelly Lima Gadelha, Kerolayne de Melo Nogueira, Álvaro Xavier Franco, Carlos Eduardo da Silva Monteiro, Maria Klayre Araújo de Sousa, Monalisa Cássias Souza and Marco Antonio de Freitas Clementino: investigation; methodology; data curation; formal analysis; validation; writing—review & editing. Lucas Antonio Duarte Nicolau, Pedro Jorge Caldas Magalhães, Alexandre Havt, Pedro Marcos Gomes Soares and Daniel Sifrim: data curation; formal analysis; validation; resources; writing—review & editing. Marcellus Henrique Loiola Ponte de Souza: conceptualization; project administration; supervision; resources; validation; data curation; formal analysis; writing—original draft; writing—review & editing; funding acquisition. All authors contributed to the study design, data acquisition/analysis, and manuscript drafting or critical revision. All approved the final version, accept accountability for the accuracy and integrity of the work and meet the official authorship criteria.

## CONFLICT OF INTEREST

None declared.

## GENERATIVE AI STATEMENT

The authors confirm that no generative artificial intelligence (AI) tools were used at any stage of the preparation, writing, revision or editing of this manuscript.

## Data Availability

Data will be made available upon request.
